# Supplementary material for: Cordycepin Resensitizes T24R2 Cisplatin-Resistant Human Bladder Cancer Cells to Cisplatin by Inactivating Ets-1 Dependent MDR1 Transcription
Source: Int J Mol Sci. 2020 Mar 2;21(5):1710. doi: 10.3390/ijms21051710 (PMC7084876; doi:10.3390/ijms21051710)
Supplement: Supplementary file 1 [file ijms-21-01710-s001.pdf]

Supplementary Materials

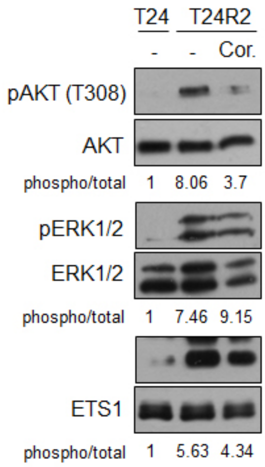

Figure S1. Cordycepin inhibits active phosphorylation of AKT. The relative ratios of phosphorylated form to total form have been determined in Image J.
